# Novel dihydroartemisinin derivative Mito-DHA_5_ induces apoptosis associated with mitochondrial pathway in bladder cancer cells

**DOI:** 10.1186/s40360-021-00542-6

**Published:** 2022-01-20

**Authors:** Linfan Xiao, Cangcang Xu, Peiyu Lin, Lingli Mu, Xiaoping Yang

**Affiliations:** Key Laboratory of Study and Discovery of Small Targeted Molecules of Hunan Province, Department of Pharmacy, School of Medicine, Hunan Normal University, Changsha, Hunan China

**Keywords:** Dihydroartemisinin derivative, ROS, MMP, Apoptosis, Bladder cancer

## Abstract

**Background:**

Bladder cancer is the second most common genitourinary malignancy and the eleventh most common cancer worldwide. Dihydroartemisinin (DHA), a first-line antimalarial drug, has been found to have potent antitumor activity. In our previous study, a novel dihydroartemisinin derivative Mito-DHA_5_ synthesized in our laboratory has a stronger anti-tumor activity than DHA. In this study, we investigated the apoptotic effect of Mito-DHA_5_ on bladder cancer T24 cells and molecular mechanisms underlying.

**Methods:**

Antitumor activity *in vitro* was evaluated by MTT, wound healing and cloning formation assays. Mitochondrial membrane potential (MMP) was detected by JC-1 probe and ROS levels were measured by specific kit. The expression of caspase-3, cleaved-caspase3, mitochondrial Cyt-C, Bcl-2, Bax and PARP in T24 cells was evaluated by Western blotting.

**Results:**

The results showed that Mito-DHA_5_ reduced cell viability with an IC_50_ value of 3.2 µM and induced T24 cell apoptosis in a dose-dependent manner, increased the production of ROS and decreased MMP. Mito-DHA_5_ could down-regulate the expression of Bcl-2, mitochondrial Cyt-C, Caspase-3, PARP and up-regulate the expression of Bax and cleaved Caspase-3.

**Conclusions:**

These data suggested that Mito-DHA_5_ had a potent inhibitory effect on T24 bladder cancer cell growth and induced these cells apoptosis associated with mitochondrial pathway.

## Background

Bladder cancer is the second most common genitourinary malignancy and the eleventh most common cancer worldwide [1]. According to the global cancer data, there are 500,000 new bladder cancer cases and 200,000 deaths in the world every year [2]. Generally, transurethral resection has served as the standard treatment. However, recurrence and metastasis are often seen in clinic after this surgery [3]. Thus, intravesical chemotherapy or immunosuppressive agents are applied to treat these patients to prevent these severe events in the commonest way [4]. However, these agents have considerable side effects such as bone marrow suppression and allergic reactions [5]. Therefore, there is an urgent need to develop anticancer agents with high efficacy and low toxicity to treat bladder carcinoma.

**Fig. 1:**
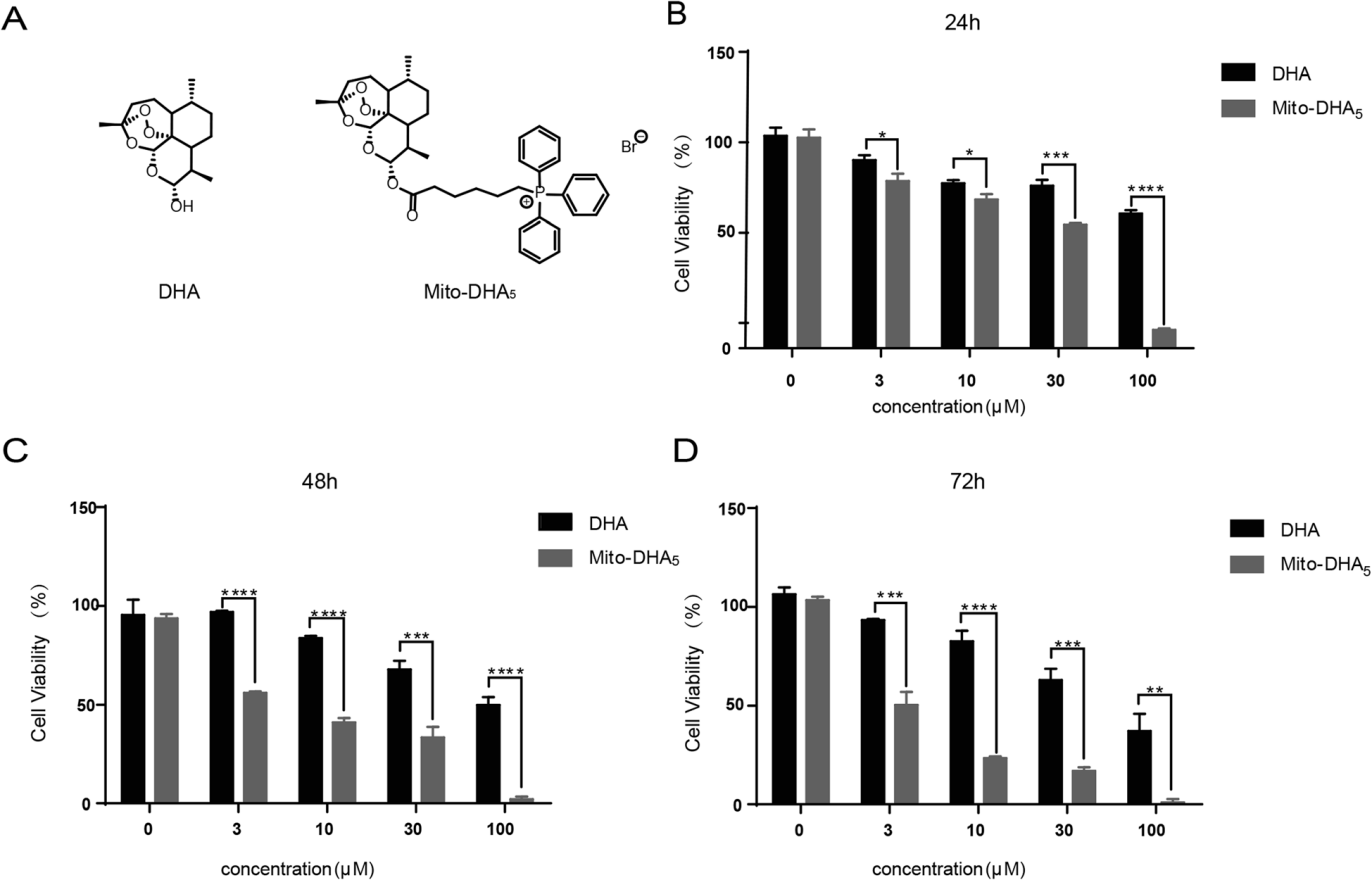
Anti proliferation activities of Mito-DHA_5_ and DHA on bladder cancer T24 cells. **A** The structures of DHA and Mito-DHA_5。_(**B**)(**C**)(**D**). T24 cells were treated with 0, 3, 10, 30, 100 µM of Mito-DHA_5_ or DHA for 24 h, 48 h, and 72 h, then observed the cell viability. Data are presented as mean±SD. *P<0.5, **P<0.01, ***P<0.001, **** *P*<0.0001

DHA is an active metabolite of artemisinin which is widely used to treat malaria in clinic [6]. Recent years, increasing number of studies reported that DHA exhibited anti-cancer activities in different kind of cancers, such as ovarian cancer [7], lung cancer [8], esophageal cancer [9], prostate cancer [10] and colon cancer [11], etc. In contrast, chemical modification of DHA is becoming a notably research area to find novel small molecules to conquer cancer. In our previous study, we found that Mito-DHA_5_ (Fig. 1 A), which is a mitochondria-targeted derivative of DHA has more potent anti-tumor activity than DHA [12]. However, the mechanisms of action of Mito-DHA_5_ remain unknown.

Mitochondria are important bioenergy factories for normal cell function and human health. In tumor cells, mitochondria are dysfunctional and cannot release apoptosis signals in time, leading to indefinite proliferation and apoptotic resistance [13]. The mitochondrial apoptosis pathway takes mitochondrial depolarization as a starting point, which is regulated by members of the Bcl-2 protein family, following the release of apoptosis signal, then activates caspase-3 to trigger apoptosis [14]. Interestingly, Farhad Poupel et al. found that DHA could induce apoptosis through mitochondrial signaling pathway in bladder cancer [15]. In order to clarify the potential mechanisms of action of Mito-DHA_5_, the effect of Mito-DHA_5_ on T24, one of representative bladder cancer cell lines, was investigated in this study.

## Materials and methods

DHA was purchased from Energy Chemical Reagent Company (Shanghai, China). Mito-DHA_5_ was synthesized in our laboratory. Compounds were dissolved in dimethyl sulfoxide (DMSO). During the experiment, the concentration of DMSO did not exceed 0.1%. Dulbecco’s modified Eagle’s medium (DMEM, U.S.), fetal bovine serum (Hyclone, Logan, UT, USA), penicillin-streptomycin solution, 0.25% trypsin and phosphate buffer (Hyclone, USA) were bought from Hyclone company. MMP Detection Kit was bought from Solarbio company (Beijing, China). AnnexinV- FITC/PI Apoptosis Detection Kit was purchased from Vazyme (Nanjing, China). ROS Detection Kit, Hochest33258 Kit, Bax (AF0057) and PARP protein antibodies were bought from Beyotime company (Shanghai, China). Bcl-2 (CAS7511) protein antibody was bought from Bioworld. Caspase-3 (#9662) and Cytochrome C (Cyt-C, D18C7) antibodies were purchased from Cell Signaling Technology company.

### Cell culture

Human bladder cancer cell T24 was obtained from Dr. P Guo (Xi’an Jiaotong University). The cells were grown in DMEM contained with 10% of FBS and 1% of penicillin–streptomycin at 37 °C, in a constant temperature incubator containing 5% of CO_2_.

### MTT assay

Briefly, T24 cells were planted in 96 well plates (8.0 × 10^3^ per well). After 12 h, treated T24 cells with different concentrations (0, 3, 10, 30 and 100 µM) of Mito-DHA_5_ or DHA for 24, 48 or 72 h. The medium was removed after 4 h incubation with MTT (2 mg/ml, 50 µL) at 37℃ and 150 µL DMSO was added. Finally, OD values were measured at 490 nm by using a microplate reader (Biotek).

### Clonogenic assay

In brief, T24 cells were planted in 24 well plates (3 × 10^3^ cells per well). After 12 h, T24 cells were treated with different concentrations (0, 1, 2, 4 µM) of Mito-DHA_5_ or DHA for about 7 days. T24 cells were fixed with 10% formaldehyde for 1 h and then 0.1% crystal violet was added to stain cells for 12 h. Finally, the OD values were measured by using an area scanning microplate reader at 550 nm.

### Wound healing assay

Briefly, T24 cells were planted in 12-well-plates (5 × 10^5^ cells per well). After the density of cells reached 90%, we created a wound on the monolayer of cell by using a 200 µL pipette tip. The floating cells were washed off by PBS for three times. Then, different concentrations (0, 1, 2, 4 µM) of Mito-DHA_5_ and DHA which were dissolved in serum-free medium were added. Finally, the scratch width of 0, 24, 48, 72 h were recorded and imaged by fluorescence microscope (DFC450C; Leica, Wetzlar, Germany).

### Hoechst 33,258 staining

Briefly, T24 cells were planted in 24 well plates (2.0 × 10^5^ cells per well). After 12 h, T24 cells were treated with Mito-DHA_5_ at 30 µM or DHA at 30 µM for 48 h. In order to investigate the effect of NAC on Mito-DHA_5_-induced apoptisis, pretreated T24 cells by 5 mM NAC for 2 h and incubated in presence of Mito-DHA_5_ (0, 10 µM) for 48 h. Then T24 cells were washed three times with PBS and stained with Hoechst 33,258 for 5 min. After three times washing with cold PBS, the morphology apoptosis of T24 cells were observed by fluorescence microscope (DFC450C; Leica, Wetzlar, Germany).

### Cell apoptosis determination

In brief, T24 cells were plated in 6-well plates (5 × 10^5^ cells per well). After 12 h, T24 cells were treated with Mito-DHA_5_ (0, 3, 10, 30 µM). After 48 h, cells were washed with cold PBS for three times and resuspended in binding buffer. Then 5 µL annexin V-FITC and 5 µL PI solution were added for staining and incubated at room temperature for 10 min in the dark. Finally, the apoptosis rate of T24 was tested with a flow cytometry (Becton Dickinson).

### Measurement of intracellular ROS generation

Briefly, T24 cells were planted in 12-well plates (3 × 10^5^ cells per well). After 12 h, T24 cells were treated with Mito-DHA_5_ (0, 3, 10 µM) or pretreated by 5 mM NAC and then cultured by Mito-DHA_5_ (0, 3 and 10 µM) for 12 h. Next removed the culture medium and then incubated with 10 µM DCFH-DA at 37 °C for 30 min. After that, washed with serum-free medium for three times and then photographed by a fluorescence microscopy (DFC450C; Leica, Wetzlar, Germany).

### JC-1 assay

MMP was detected using JC-1 staining kit. In brief, T24 cells were plated in 12-well plates (2.0 × 10^5^ cells per well). After 12 h, T24 cells were treated with different concentrations (0, 3, 10 and 30 µM) of Mito-DHA_5_ or DHA (30 µM) for 48 h. First, prepared incubated JC-1 stain working solution according to the instruction. Briefly, added 50 µL JC-1 (200X) to 8 mL super-pure water and mixed fully. Then 2 mL JC-1 staining buffer (5X) was added to it. After the completion of drug treatment, T24 cells were stained with JC-1 stain working solution (0.5 mL) at 37 °C for 20 min. Finally, washed with JC-1 staining buffer (1×) for twice and added 2 mL incubation medium. Fluorescence microscopy was used to measure MMP.

### Western blotting analysis

Briefly, T24 cells were planted in 6-well-plates (5 × 10^5^ cells per well). After 12 h, T24 cells were treated with Mito-DHA_5_ (0, 3, 10 and 30 µM). After 24 h, washed with cold PBS for twice and lysate was added for 30 min on ice. Then samples were cooked in boiling water for 10 min immediately and stored at -20 ℃. Western blotting experiment was carried out with regular procedure. In brief, PVDF membrane was blocked in 5% milk for 1 h after eletrophoresis finished. Then incubated in first antibody at 4℃. After 15-18 h, washed with PBST for three times (10 min per time) and then incubated in secondary antibody for 1 h at room temperature. After that, washed with PBST for three times again. Finally, developing solution was added and imaged on Chemi Doc (Bio-Rad, USA).

### Data analysis

We used the GraphPad Prism (version 6.01) software to analyze our data and the results were expressed as the mean ± SD. The statistical significance of the data was analysed by T-test and was indicated as: *p<0.05, **p<0.01, ***p<0.001 and **** *p*<0.0001 ns: represent no significance.

## Results

### Mito-DHA_5_ inhibits the proliferation of T24 cancer cell line

The structures of Mito-DHA_5_ and DHA were shown in Fig. 1 A. The anti-proliferation activities of Mito-DHA_5_ and DHA in T24 cells were assessed by MTT assay. As shown in Fig. 1B, C and D, incubated T24 cells with different concentrations of Mito-DHA_5_ for 24 h, 48 and 72 h resulted in significant reduction of the cell viability compared with DHA in a dose-dependent manner and time-dependent manner. In order to evaluate the potency, half maximal inhibitory concentration (IC_50_) value of 72 h for T24 cell line was calculated for Mito-DHA_5_ (3.2±0.74 µM) and DHA (71.5±5.24 µM), respectively (Table 1). Mito-DHA_5_ displayed 22 times higher inhibitory effect than DHA in the T24 cancer cell line.

**Table 1:** The anti-proliferation activity of Mito-DHA_5_ and DHA against T24 cell line

Compounds	IC_50_ (µM, 72 h)
	T24
Mito-DHA_5_	3.2±0.74
DHA	71.5±5.24

### Mito-DHA_5_ inhibits colony formation of T24 cells

**Fig. 2:**
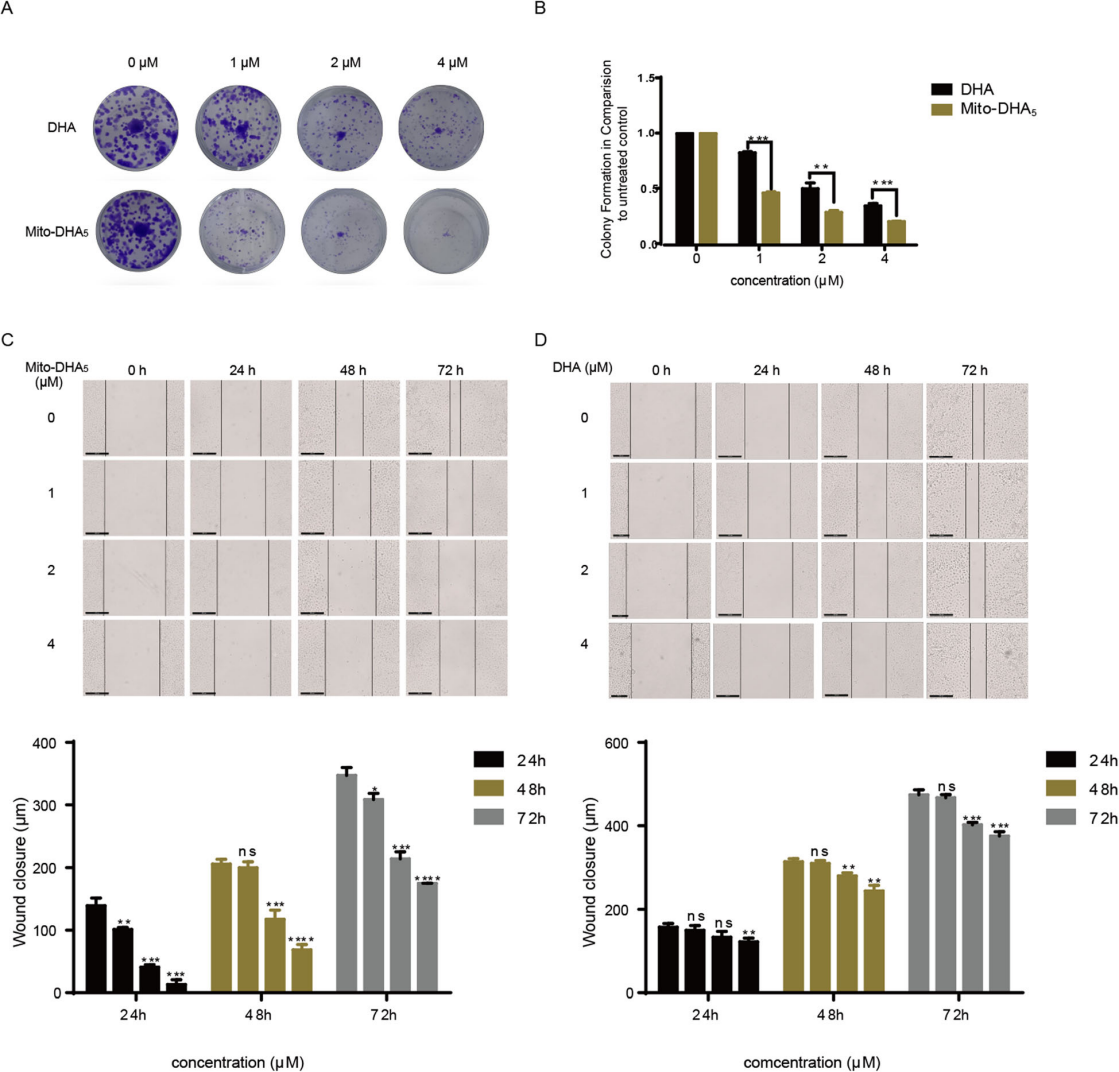
The inhibition of colony formation and migration of Mito-DHA_5_ and DHA on T24 cells. **A** Evaluation of colony suppression by Mito-DHA_5_ and DHA. **B** Quantification of the colony formation. OD values were scanned at a wavelength of 550 nm. **C** Inhibitory migration effect of Mito-DHA_5_ on T24 cells for 24, 48, 72 h (**D**). Inhibitory migration effect of DHA on T24 cells for 24, 48, 72 h. Scale bar was 200 μm. Data are presented as mean±SD. *P<0.5, **P<0.01, ***P<0.001, **** *P*<0.0001, ns: no significance

The effect of Mito-DHA_5_ in inhibiting the clonogenicity of T24 cells was evaluated. The results showed that Mito-DHA_5_ exerted potent inhibitory effect on T24 cells (Fig. 2 A). At 4 µM of Mito-DHA_5_, almost no colony formation was observed. The effect was dose-dependent and much more potent than the DHA treatment group at the same concentration (Fig. 2B).

### Mito-DHA_5_ inhibits migration of T24 cells

The effect of Mito-DHA_5_ in inhibiting the migration of T24 cells was evaluated. As shown in Fig. 2 C, after the treatment of T24 cells with different concentrations of Mito-DHA_5_, cell migration was significantly inhibited at 24, 48 and 72 h compared with control. However, DHA did not obviously inhibit the migration of T24 cells at the same concentration (Fig. 2D). These results indicated that Mito-DHA_5_ has stronger inhibitory effect than DHA on the migration of T24 cells.

### Mito-DHA_5_ induces cell apoptosis in T24 cells

**Fig. 3:**
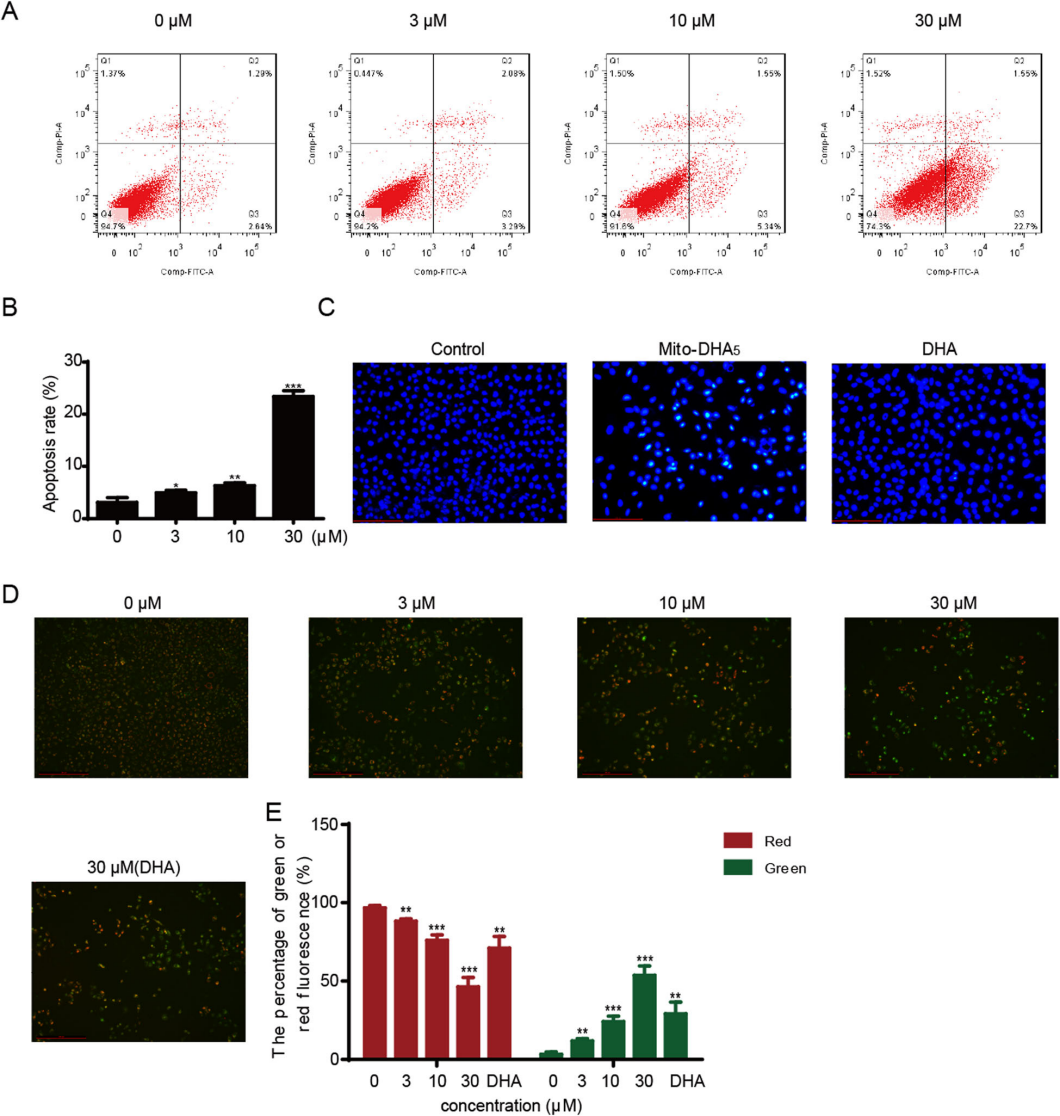
Effect of Mito-DHA_5_ on cell apoptosis and MMP in T24 cells. **A** T24 cells were treated with Mito-DHA_5_ (0, 3, 10, 30 µM) for 48 h and then assayed by flow cytometry analysis with Annexin V-FITC staining. **B** Quantification of apoptotic cells. **C** Treat T24 cells with 30 µM of Mito-DHA_5_ and DHA for 48 h, respectively, stained with Hochest 33,258, and viewed by fluorescence microscopy. Representative images were shown. Scale bar was 100 μm. **D** T24 cells were treated with Mito-DHA_5_ (0, 3, 10, 30 µM) or DHA (30 µM) for 48 h and then viewed by fluorescence microscopy after JC-1 staining. Increased green fluorescence represents a decrease in MMP. Scale bar was 200 μm. **E** The percentage of green fluorescence and red fluorescence. Results are the mean±SD of three independent experiments. *P<0.5, **P<0.01, ***P<0.001, **** *P*<0.0001

We used annexin-V and PI double staining to determine whether the growth-inhibiting effect of Mito-DHA_5_ in T24 cells was related to cell apoptosis. After the treatment of Mito-DHA_5_ with different concentrations for 48 h, we analyzed the results by flow cytometry. As shown in Fig. 3 A, Mito-DHA_5_ induced significantly cell early apoptosis in T24 cells in a dose-dependent manner. Total apoptosis rate at 30 µM was 24.2% (Fig. 3B). In order to explore whether the effect of Mito-DHA_5_ in inducing cell apoptosis was stronger than DHA, the nuclei change in T24 was observed under a fluorescence microscope by Hoechst 33,258. As shown in Fig. 3 C, treatment with Mito-DHA_5_ at 30 µM induced more significant bright blue nuclei blebbing, nuclear rounding and shrinkage than treatment with DHA at 30 µM in T24 cells. And the cell density of treatment with Mito-DHA_5_ under the same concentration was much lower than that of treatment with DHA.

### Mito-DHA_5_ decreases MMP in T24 cells

To explore the effect of Mito-DHA_5_ in MMP in T24 cells, we used the JC-1 staining method. After the treatment of Mito-DHA_5_ with different concentrations for 48 h, the red fluorescence and green fluorescence were analyzed. An increase in green fluorescence intensity represents a decrease in MMP, while the red fluorescence intensity represents an increase in MMP. As shown in Fig. 3D and E, Mito-DHA_5_ caused a decrease in MMP of bladder cancer T24 cells with a dose-dependent fashion. Compared with the ratio of green fluorescence/red fluorescence of DHA at 30 µM, Mito-DHA_5_ increased the ratio at the same concentration, indicating the stronger effect of Mito-DHA_5_ on decreasing capacity of MMP.

### Mito-DHA_5_ increases the ROS level in T24 cells

**Fig. 4:**
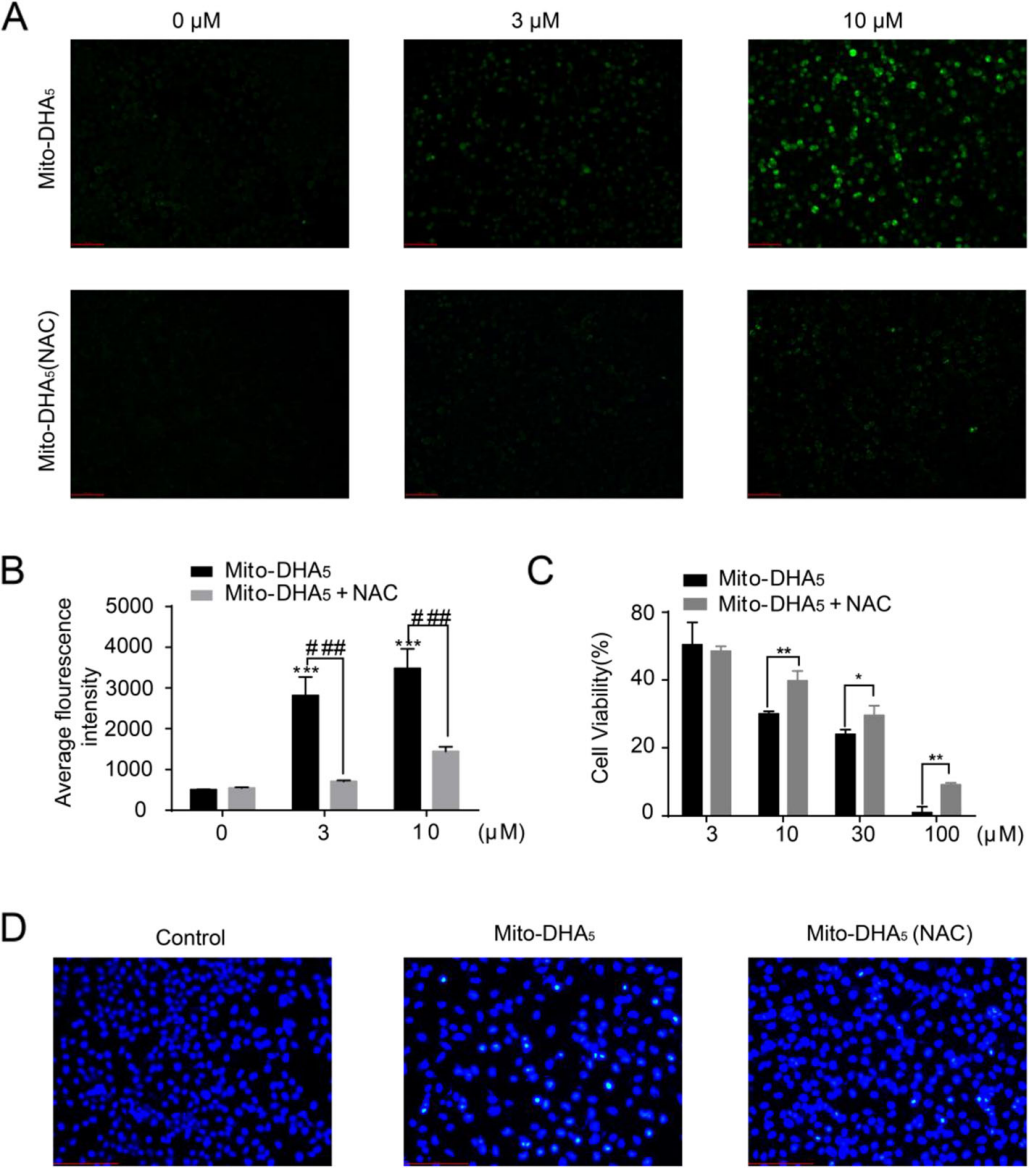
Effects of Mito-DHA_5_ on ROS production. **A** T24 cells were treated with Mito-DHA_5_ (0, 3, 10 µM) in the presence or absence of pretreated with 5 mM NAC for 24 h, then DCFH-DA (10 µM) was loaded and cells were analyzed by fluorescence microscopy. Scale bar was 100 μm. **B** Average fluorescence intensity of different treatment groups. **C** Effects of NAC on Mito-DHA_5_-induced cytotoxicity in T24 cells assessed by MTT. Cells were treated with Mito-DHA_5_ (3, 10, 30, 100 µM) for 72 h in the presence or absence of pretreated with 5 mM NAC. **D** T24 Cells were treated with Mito-DHA_5_ (10 µM) in the presence or absence of pretreated with 5 mM NAC for 48 h, stained with Hochest 33,258, and viewed by fluorescence microscopy. Scale bar 100 was µm. Data are presented as mean ±SD. *P<0.5, **P<0.01, ***P<0.001, ^#^*P*<0.5, ^##^*P*<0.01, ^###^*P*<0.001

We evaluated how treatment with Mito-DHA_5_ in T24 cells influences the production of ROS tracked by DCFH-DA. Mito-DHA_5_ induced an increase of ROS production in a dose-dependent manner and the pretreatment of NAC significantly reduced ROS production (Fig. 4 A and 4B). The cytotoxicity of Mito-DHA_5_ on T24 cells could be inhibited by NAC (Fig. 4 C). The cell morphologic change was observed by fluorescence microscopy. The results showed that with the pretreatment of NAC, the induction of cell apoptosis was inhibited in T24 cells (Fig. 4D), indicating that Mito-DHA_5_ induced T24 cell apoptosis dependent on ROS.

### Apoptosis effect of Mito-DHA_5_ was associated with mitochondrial pathway

**Fig. 5:**
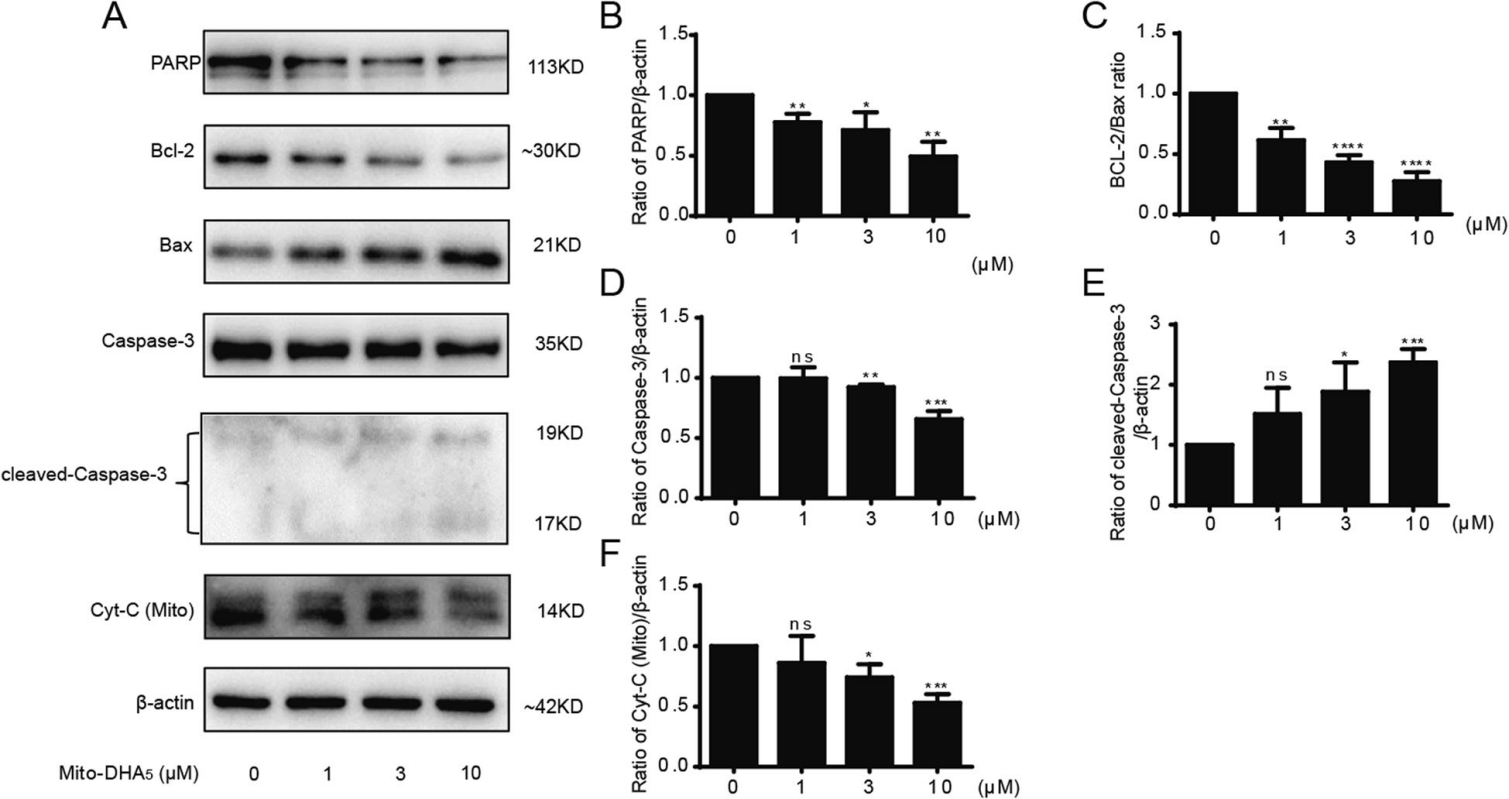
**A** The expression level of cell apoptosis regulatory proteins Bcl-2, Bax, Caspase-3, cleaved-Caspase3, mitochondrial Cyt-C and PARP in T24 cells after treatment Mito-DHA_5_ (0, 1, 3, 10 µM). **B** The expression level of PARP protein. **C** The ratio of BCL-2/Bax protein. **D** The expression level of Caspase-3 protein. **E** The expression level of cleaved-Caspase3 protein. **F** The expression level of mitochondrial Cyt-C protein. Data are presented as the mean±SD of three independent experiments. *P<0.5, **P<0.01, ***P<0.001, **** *P*<0.0001, ns: represent no significance

To further investigate whether Mito-DHA_5_ influences the apoptosis-related protein expression (Fig. 5), Western blotting was used to detect the levels of mitochondrial pathway associated proteins. After the treatment of T24 cells with different concentrations of Mito-DHA_5_ (0, 1, 3 and 10 µM) for 24 h, we found that Mito-DHA_5_ treatment could down-regulate the expression of Bcl-2 and up-regulate the expression of Bax in a dose-dependent manner. At the same time, Mito-DHA_5_ could down-regulate the expression of mitochondrion Cyt-C and further activate caspase-3. Finally, increased the expression of cleaved caspase-3 and down-regulate the expression of PARP.

## Discussion

Mito-DHA_5_ is a newly synthesized mitochondrial-targeted artemisinin ester derivative. Our previous studies have found that this compound has great anti-tumor activity [12]. In this study, we have provided evidence that Mito-DHA_5_ induced mitochondria-associated apoptosis, decreased MMP, increased ROS level, and leaded to caspase-3 activation associated with mitochondrial-dependent apoptosis pathway in T24 bladder cancer cells.

Apoptosis is a type of programmed cell death. This programmed cell death process is mediated by a variety of signal pathways (internal and external) triggered by a variety of factors (including cell stress, DNA damage, and immune monitoring) [16, 17]. Recent studies reported that DHA can significantly induce apoptosis of tumor cells [18]. Haiting Mao et al. found that mitochondrial pathway played an important effect in the process of DHA-induced breast cancer cell apoptosis [19]. Another study reported that DHA induced apoptosis through activation of JNK1/2 and p38 MAPK signaling pathway in human gastric cancer cell line BGC-823 [20]. In this study, we evaluated the ability of Mito-DHA_5_ to induce apoptosis of T24 bladder cancer cells. The ratio of apoptosis cells reached 24.2% after incubation with Mito-DHA_5_ of 30 µM in T24 cells. At the same concentration, Mito-DHA_5_ had a stronger ability to induce cell apoptosis than DHA. MMP is closely related to cell apoptosis, and the decrease of MMP indicates that cells may undergo early apoptosis [21]. We treated T24 cells with Mito-DHA_5_ of different concentrations, and found that Mito-DHA_5_ could significantly reduce the MMP at the concentration of 30 µM. In addition, the MMP of the DHA treatment group under the same concentration less than that of the Mito-DHA_5_ treatment group. This result was consistent with the apoptosis result, and further told us that Mito-DHA_5_ could induce apoptosis of T24 cells with much stronger apoptosis than that of DHA.

Reactive oxygen species (ROS) are a family of short-lived molecules and the production of ROS participates in radiotherapy by affecting downstream cell death signals [22, 23]. Mitochondria, the place where cells breathe, are the main place where endogenous ROS is produced. A sharp increase in ROS in a short period of time will lead to the occurrence of cell apoptosis [24]. Our study showed that Mito-DHA_5_ could cause an increase in the level of ROS in T24 cells, and this increase could be inhibited by the antioxidant NAC. At the same time, the killing effect of Mito-DHA_5_ on T24 cells was weakened under the condition of NAC pretreatment. Besides, the results of fluorescence microscopy showed that the apoptosis ratio of the NAC pretreatment group decreased significantly under the same concentration of Mito-DHA_5_ treatment. This implied us that Mito-DHA_5_-induced apoptosis might be related to the production of ROS.

The Bcl-2 family plays an important role in cell apoptosis [25]. The role of the Bcl-2 family in the regulation of apoptosis is generally described as anti-apoptotic and pro-apoptotic [26]. Bcl-2 is anti-apoptotic protein, while Bax is pro-apoptotic protein and the ratio of them is closely related to cell apoptosis. Mito-DHA_5_ treatment resulted in an obvious decrease in Bcl-2 protein expression and a significant increase in Bax protein expression in T24 cells. This indicated that Mito-DHA_5_ induced the cell apoptosis through the regulating of the ratio of Bcl-2/Bax. Cytochrome C (Cyt-C) is an electron transporting protein, upon receiving the apoptosis signal, Cyt-C is rapidly released from the mitochondrion. Cytosolic Cyt-C can activate Caspase-3 and eventually leads to apoptosis [27]. Caspase-3 is the downstream pathway of cell apoptosis and can be activated when cells apoptosis happens [28]. PARP is a protein closely related to DNA repair and is an important indicator of Caspase-3 activation. PARP can be cleaved by activated Caspase-3 [29]. Our research showed that after treating T24 cells with Mito-DHA_5_, the expression of mitochondrial Cyt-C was decreased and cleaved Caspase-3 was increased. Meanwhile, the expression of PARP was decreased. These data indicated that Mito-DHA_5_ could induce T24 cells apoptosis associated with mitochondrial-mediated pathway.

## Conclusions

In conclusion, this research demonstrated that Mito-DHA_5_ could induce apoptosis associated with mitochondrial-mediated pathway, decrease MMP, increased ROS level and resulting in down-regulation of the Bcl-2/Bax ratio and downstream activation the caspase-3. Collectively, these results suggest that Mito-DHA_5_ holds promise for further development as a candidate for the treatment of bladder cancer.

## Data Availability

All data and materials are contained and described within the manuscript.

## Abbreviations

DHA: Dihydroartemisinin
MMP: Mitochondrial membrane potential
ROS: Reactive oxygen species
DMSO: Dimethyl sulfoxide
DMEM: Dulbecco’s modified Eagle’s medium
MTT: 3-(4, 5-dimethylthiazol-2-yl)–2, 5- diphenyltetrazolium bromide
